# Trusting the Psychotherapist, Trusting the Governmental System and Perhaps Trusting the Neighbor Next Door: Exploring the Institutional Nature of Trust in Psychotherapists and Examining Different Groups of Trusters in a Large German Patient Sample

**DOI:** 10.32872/cpe.19731

**Published:** 2026-08-31

**Authors:** Marie-Theresa Kaufmann, Christoph Kasinger, Ayline Heller, Elmar Brähler, Bernhard Strauß

**Affiliations:** 1 Institute for Psychosocial Medicine, Psychotherapy and Psychooncology, Friedrich Schiller University Jena, Jena, Germany; 2 Department of Psychosomatic Medicine and Psychotherapy, University Medical Center, Johannes Gutenberg University Mainz, Mainz, Germany; 3 Survey Design and Methodology, GESIS – Leibniz-Institute for the Social Sciences, Mannheim, Germany; 4 Department of Medical Psychology and Medical Sociology, University of Leipzig, Leipzig, Germany; Philipps-University of Marburg, Marburg, Germany

**Keywords:** trust in psychotherapists, psychotherapy, systematic change, institutional trust, interpersonal trust

## Abstract

**Background:**

Trust in psychotherapists is considered primarily interpersonal. However, psychotherapists could be viewed as representatives of the (healthcare) system. In this study, we investigated whether trust in psychotherapists is more closely linked to interpersonal or institutional trust. Furthermore, we explored whether people can be distinguished into different trust groups.

**Method:**

In a German cross-sectional study sample, we identified 1,300 persons (61.7% female; *M* = 48.46 years; *SD* = 14.45 years) indicating experiences with psychotherapy (current and/or in the past). Correlation and linear regression analysis were applied to explore connections between interpersonal (SOEP Interpersonal Trust Scale), institutional (ALLBUS Institutional Trust Scale), and trust in psychotherapists (IIV Psychotherapists). Then, hierarchical cluster analysis was used to identify profiles of interpersonal, institutional, and trust in psychotherapists. Group differences of the cluster solution were analyzed for dimensions that are important for trust and psychotherapy such as having experienced trauma.

**Results:**

Trust in psychotherapists is not solely associated with interpersonal factors but is also modestly associated with institutional trust. Furthermore, a three-cluster solution emerged with different trust profiles: *low trusters* (30%) with low scores for all trust measures, *high trusters* (49%) with the highest values, and *system trusters* (21%) with the highest trust scores in psychotherapists and institutions, but lower interpersonal trust values. The three groups showed different manifestations of e.g., trauma (LEC-5 Personal Experienced Subscale).

**Conclusion:**

There is an institutional nature of trust in psychotherapists, which should be reflected and included in research and practice. Implications could include the common measurement of trust in psychotherapists in therapy and broadening psychotherapeutic interventions according to trust needs. To this end, new ways of building trust both in psychotherapy and institutions in general should be explored.

## Highlights

- Trust in psychotherapists should be considered partly institutional in nature.
- Trust in psychotherapists is associated with both interpersonal and institutional trust.
- Three distinct trust profiles emerged: low trusters, high trusters, and system trusters.

“[T]rust (or, symmetrically, distrust) is a particular level of the subjective probability with which an agent assesses that another agent or group of agents will perform a particular action, both before he can monitor such action” (Gambetta, 2000, p. 216). Understanding trust in psychotherapists is crucial for assessing impact factors of psychotherapy (e.g., Gaebel et al., 2014; Strupp et al., 1964). Although trust in psychotherapists is often discussed as interpersonal (e.g., Kassebaum, 2004; Ozawa & Sripad, 2013), there is limited evidence to support this assumption. As psychotherapists operate within the public institution ‘healthcare system’, connections with institutional trust are conceivable. A comprehensive understanding of the interrelationships between trust in psychotherapists, institutions, and interpersonal trust is essential for developing effective approaches to fostering trust in psychotherapy.

## Trust in Psychotherapists

Trust in psychotherapists plays a key role in help-seeking behavior (Cavanagh et al., 2022). It is also strongly linked to better treatment outcomes, particularly when patients regularly communicate with their psychotherapist about whether they feel respected and trust in their relationship (Connolly Gibbons et al., 2023). Trust ratings of psychotherapists in their patients and vice versa do not necessarily have to correlate, but therapists’ evaluations of being congruent and empathic correlate with patients’ trust ratings (Peschken & Johnson, 1997). It remains questionable whether one can really speak of interpersonal trust between patient and practitioner. It may be a question of trust between two representatives of two groups who meet (Sousa‐Duarte et al., 2020).

Trust measures can either refer to psychotherapists in general or to one's own psychotherapist with whom one is undergoing treatment. While there is a larger number of survey instruments available for measuring trust in one's own treating psychotherapist, which is a time critical event that must be completed during psychotherapy (e.g., Crits-Christoph et al., 2019), there is a lack of questionnaires that focus on general trust in psychotherapists (Kassebaum, 2004), which this study investigated.

## Interpersonal Trust

The concept of interpersonal trust is understood as trust in strangers or trust in people in general (Beierlein et al., 2012; Naef & Schupp, 2009; Rotter, 1967; Yamagishi & Yamagishi, 1994). There are different definitions, which contain (a) a subject, (b) an action, and (c) an expectation concerning the future. “Trust, however, involves present decisions, often based on another person’s past behavior, that require anticipating some action that hasn’t yet happened” (Borum, 2010, p. 8). Lower levels of interpersonal trust are associated with reduced economic performance as lower income or higher rates of unemployment (Lichter et al., 2021; Nikolova et al., 2022). Individuals with higher interpersonal trust values are happier and more socially integrated (Rotter, 1980). Furthermore, greater interpersonal trust is linked to higher quality of life (Tokuda et al., 2008) and better mental health (Folkman et al., 1986).

## Institutional Trust

Institutional trust is typically measured using scales that assess the level of trust in specific institutions (Hudson, 2006; Schupp & Wagner, 2004), which are evaluated by residents of a country (González & Smith, 2017). Institutional trust can be separated into three categories: political trust (e.g., in the parliament), trust in impartial institutions (e.g., the healthcare system), and trust in control institutions (e.g., the media). Among these, the healthcare system generally achieves the highest scores in comparison with other institutions, such as the media (Rothstein & Stolle, 2002). Trust is also a crucial factor in both private and social contexts, which are highly relevant for psychotherapy: Higher levels of institutional trust have been linked to enhanced social cohesion and mental health outcomes (Defeyter et al., 2021; van Prooijen et al., 2022). Moreover, institutional trust can act as a protective factor against the negative impact of adverse life events on well-being (Glatz & Eder, 2020; Lee, 2022). It also appears to play a significant role in the economic management of a country and for coping with crises (Chen et al., 2024; Eitze et al., 2021; Lichter et al., 2021; Reinemann et al., 2022).

## Trust in Psychotherapists, Interpersonal Trust, and Institutional Trust – Considerations About Connections

Studies on the measurement of trust in (healthcare) systems, healthcare practitioners and the trust between practitioners, and patients primarily rely on questionnaires focused on the patient-practitioner relationship (Aboueid et al., 2023; Ozawa & Sripad, 2013). Thereby, these instruments neglect to address system trust (Ozawa & Sripad, 2013).

Notably, interpersonal and institutional trust appear to be distinct concepts as they differ according to their relationship to other constructs and attitudes (Krastev et al., 2023) and can be distinguished in factor analysis (Naef & Schupp, 2009). A causal influence of institutional trust on interpersonal trust has been proposed (Sønderskov & Dinesen, 2016). On the other hand, interactions with practitioners are still considered an interpersonal factor in the development of institutional trust (Campos-Castillo et al., 2016). It is important to clarify these connections as they affect the effectiveness of psychotherapy.

Several factors identified in the literature as influencing the utilization of psychotherapy overlap with those associated with higher levels of institutional trust: The sociodemographic factors such as biological sex (female), higher education, not living together with a partner, income and age appear to be relevant in understanding the likelihood of psychotherapy utilization (e.g., Hahm et al., 2020; Petrowski et al., 2014; Vessey & Howard, 1993). Similarly, the sociodemographic factors gender, high school diploma, age and income have been identified as significant factors for institutional trust (Baroudi et al., 2022; Campbell, 2004; Campbell, 2023). This may indicate that there are the same influencing factors and possibly conceptual similarities of trust in psychotherapists and institutions. Since the present cross-sectional study design is exploratory, further relationships will be recorded here. Psychotherapy is embedded within the healthcare system and is regulated by institutional frameworks in Germany. As such, trust in psychotherapists may not only reflect interpersonal evaluations but also institutional trust that governs and provides psychotherapy.

Linking trust in psychotherapeutic settings to institutional trust could offer valuable insights into the factors influencing therapy effectiveness and accessibility. Understanding this connection can help identify barriers to treatment, improve patient engagement, and highlight the broader societal impact of institutional trust on health behaviors.

In this study, we aimed to investigate the relationships between the three different forms of trust: in psychotherapists, others, and institutions. Based on the literature, we assumed a stronger connection of trust in psychotherapists to institutional trust than to interpersonal trust.

Patients come to therapy with varying backgrounds, including experiences of trauma, resilience, personality traits, and beliefs. For this reason, the results were further contextualized in terms of factors relevant to psychotherapy: Associations between trust patterns and traumatic life events (Goenjian et al., 1997) were also investigated, as they contribute to psychological burden and psychotherapy usage. Differences in psychological resilience factors, such as general resilience (Bartholomew et al., 2022), belief in a just world (Furnham, 2003), and willingness to forgive others – a resilience factor for traumatic life events (Wade et al., 2005) – were examined. Personality traits were also analyzed as introversion and neuroticism may be risk factors for psychological disorders (Zinbarg et al., 2008).

## Method

### Study Design, Setting, and Participants

An online cross-sectional study was conducted in Germany in December 2022 by two projects^1^1The two projects ‘SiSaP’ (University Hospital Jena) and ‘DDR-Psych’ (University Hospital Mainz) investigated the history of psychotherapy in the GDR (German Democratic Republic) and the psychological consequences of the former GDR., which were funded by the German ‘Federal Ministry of Research, Technology and Space’ (‘Bundesministerium für Forschung, Technologie und Raumfahrt’; BMFTR). The study was approved by the Ethics Committee of the University Hospital Jena (Reg.-Nr. 2022-2817-Bef). The analyzed questions were part of an extensive survey in which a series of health-related and psychological data were collected. Around 2000 East Germans, 2000 West Germans, and 500 internal migrants from East to West Germany, over the age of 18 up to 74, were contacted via the panel provider bilendi & respondi. The provider sent out three separate invitations for the three groups (East Germans, West Germans, and internal migrants). For the last group, a low frequency in the population (Heller et al., 2020) was expected, and a correspondingly high number of invitations was necessary. The participating panelists were unaware that the aforementioned groups were being targeted. They began the survey and were excluded based on their feedback regarding the relevant sociodemographic factors if they did not meet these criteria. They received financial compensation. Consequently, the response rates varied between 0.1% and 9%. Quotas on gender (female/male) and age (uncrossed) were only applied to East and West Germans, not to internal migrants, due to the low frequency of internal migrants in the population. There were no missing values, as the participants could only continue and complete the questionnaire if they filled out all fields. A total number of *n* = 170 people were excluded due to unrealistic values (number of people in the household > 10; frequency of one's own unemployment > 10). This resulted in a sample of *N* = 4,619 people (Table S1 Appendix). In our analysis, only *n* = 1,300 people that experienced psychotherapy after the fall of the Berlin wall and the end of the Cold War in the year 1990 were included (Table 1). We did not consider those with psychotherapy experience before the end of the Cold War 1990, as Germany was divided into two completely different governmental systems with different healthcare systems in these decades (see section Psychotherapy Experience).

**Table 1:** Subsample Description of the Cross-Sectional Study (Online)

Variable	Participants (*n* = 1,300)
Female gender	*n* = 799 (61.7%)
Age	*M* = 48.46 (*SD* = 14.45)
Home ownership	*n* = 449 (34.5%)
Interpersonel trust*	*M* = 6.54 (*SD* = 1.63)
Trust in institutions**	*M* = 3.77 (*SD* = 1.27)
Trust in psychotherapists***	*M* = 3.86 (*SD* = 0.73)

*Note.* The relative proportions refer to the total number of the sub-sample. Reflects *M* and *SD* of participants showing trust from a scale from 3 to 12*, 1 to 7**, 1 to 5***.

### Instruments

#### Psychotherapy Experience

The psychotherapy variable (binary: yes/no) was generated based on the response to the following question: ‘*Have you ever received psychotherapy as an inpatient in a clinic or as an outpatient, without a stay in a hospital?*’ The response options were '*no*', '*yes, started and already completed*', *'yes, started and discontinued*' and *'yes, currently*'. A distinction was made between '*no*' and '*yes*' answers. For people who were born before the 1^st^ of January 1980^2^2This was a distinction measure for people that experienced the Cold War (similar to Ulke et al., 2021). the question was asked twice – once related to the Cold War period when Germany was still divided and once to the period after 1990. In conducting the calculations, only those therapeutic experiences were used that occurred subsequent to the fall of the Berlin wall and the end of the Cold War in 1990 because of system comparability. In total *n* = 1,300 received psychotherapy after the end of the Cold War.

#### Trust Questionnaires

Institutional trust was measured by the questionnaire of the German General Social Survey (ALLBUS; GESIS, 2018). It contains 13 categories such as the healthcare system, the German parliament or television and is a validated instrument (Naef & Schupp, 2009). For each category, the participant rates on a 7-point scale how much he or she trusts in it (*1 not at all*; *7 very much*) – an averaged total value between 1 and 7 was calculated. Higher values indicate higher levels of trust. The scale showed excellent internal consistency (α = .95), indicating a highly reliable measure.

Interpersonal trust was measured with the validated three item scale of the German Socio-Economic Panel Study with questions such as *“In general, you can trust people”* (SOEP; Naef & Schupp, 2009; *1 not at all; 4 very much*). Although brief, the SOEP measure is widely used in large-scale studies and provides an efficient assessment of interpersonal trust. A sum score was calculated with values ranging between 3 and 12. Higher values indicate higher levels of trust. The scale showed moderate internal consistency (α = .60), which may reflect the conceptual heterogeneity of the construct. Given the exploratory nature of the study, this level of reliability was considered acceptable.

Trust in psychotherapists was measured with the validated scale for trust in psychotherapists in Kassebaum’s (2004) questionnaire including further trust categories such as trust in neighbours which were not used. The questionnaire asks e.g., *“Psychotherapists can be a great help in serious crises”* (*1 NO - - I completely disagree with this statement.; 5 YES + + I completely agree with this statement.*). A mean value was calculated with results ranging between 1 and 5. Higher values indicate higher levels of trust. The scale demonstrated good internal consistency (α = .79), indicating a reliable measurement of the construct.

#### Classification Questionnaires for the Trust-Profiles

Potentially traumatic life events were assessed with the Life Event Checklist (LEC-5; Gray et al., 2004; Weathers et al., 2013). The questionnaire comprises 16 events, which may be experienced by the individual, witnessed or heart about by the individual or with the individual experiencing it through the job. In addition, there is the possibility of assessing uncertainty regarding whether an event has been experienced or not and also reporting being not affected. A sum score of events which happened to oneself personally was calculated.

Belief in a just world was measured with the validated questionnaire of Dalbert and colleagues (1987; Schmitt et al., 2008) comprising six items on a scale from *1* (disagreeing) up to *6* (agreeing) – indicating more belief with a higher sum score.

The willingness to forgive was measured with the validated questionnaire of Allemand and colleagues (2008). Two subscales are utilized to ascertain an individual’s willingness to forgive another person who has (not) expressed regret for their actions on a scale from *1* (not at all) up to *5* (very much) – indicating more willingness with a higher score. A total score was calculated.

Resilience was measured with the validated RS-5 scale comprising five items (Wagnild & Young, 1993; Schmalbach et al., 2016). Higher values indicate higher levels of resilience.

Personality factors were measured with the validated Big Five Inventory BFI-10 with two items for each category (openness, extraversion, agreeableness, conscientiousness and neuroticism). One item of each category is inverted. Each item had to be answered on a five-point scale from *1* (disagreeing) up to *5* (agreeing; Rammstedt & John, 2007). A mean score was calculated.

### Statistical Analysis

The analyses were carried out using the statistical software IBM SPSS (Version 21). Analyses concerning gender issues included only male and female respondents due to the small number of people who selected the category ‘diverse’ or ‘other’. Results were considered significant at α = 0.05.

#### Identification of Connections Between Interpersonal, Institutional and Trust in Psychotherapists

Correlational and linear regression analyses were applied to identify connections between the three trust scores. Correlational analysis included in the first analysis trust in psychotherapist and institutional trust and in a second analysis trust in psychotherapists and interpersonal trust. The linear regression analysis to predict trust in psychotherapists included institutional trust an interpersonal trust.

#### Identification of Pattern-Based Subcategories With Different Trust-Profiles and Validation of Trust-Profiles

Trust in psychotherapists, institutional trust, and interpersonal trust were used as input for an agglomerative hierarchical clustering algorithm (HCA). HCA used the Ward error sum-of-squares agglomeration method with Euclidean distance between individuals. Following these steps, individuals who have experienced psychotherapy were categorized into pattern-based subcategories (clusters). The number of clusters was determined by inspecting the dendrogram, with particular attention to increases in linkage distances between successive fusion steps. A marked increase in these distances suggested a three-cluster solution, indicating that further merging would substantially reduce within-cluster homogeneity. To assess the separation of the clusters, a discriminant analysis was subsequently performed. The differences between clusters were statistically significant (*p* < .001; Table S2 Appendix).

#### Classification of Trust-Profiles

Kruskal-Wallis-Tests were mainly used for the calculations, only for the Big Five personality traits openness and agreeableness were ANOVAs used. Pairwise comparisons were calculated with the Dunn test (Kruskal-Wallis-Test) and the Scheffé procedure (ANOVA).

## Results

### Identification of Connections Between Interpersonal, Institutional, and Trust in Psychotherapists

Trust in psychotherapists and institutional trust correlated on a significant level of *r* = 0.259 (*p* < .001). Trust in psychotherapists and interpersonal trust also correlated significantly *r* = 0.226 (*p* < .001). Multiple linear regression analysis (*R*^2^ = 0.089 (adj. *R*^2^ = 0.088); *F*(2, 1297) = 63.64, *p* < .001) confirmed the relevance of both, with institutional trust showing a slightly stronger connection as standardized β reached a higher value (Table 2).

**Table 2:** Multiple Linear Regression Model

Variable	Multiple linear regression model (*n* = 1,300)		
	*B* (95% CI)	β	*p*
Constate term	2.985 (2.781; 3.188)		< .001***
Interpersonal trust	0.085 (0.055; 0.114)	0.158	< .001***
Trust in institutions	0.142 (0.104; 0.180)	0.207	< .001***

*Note. B* = unstandardized coefficient; β *=* standardized coefficient.

***p < .001.

### Pattern-Based Subcategories With Different Trust-Profiles Among Participants That Have Experienced Psychotherapy

**Figure 1:**
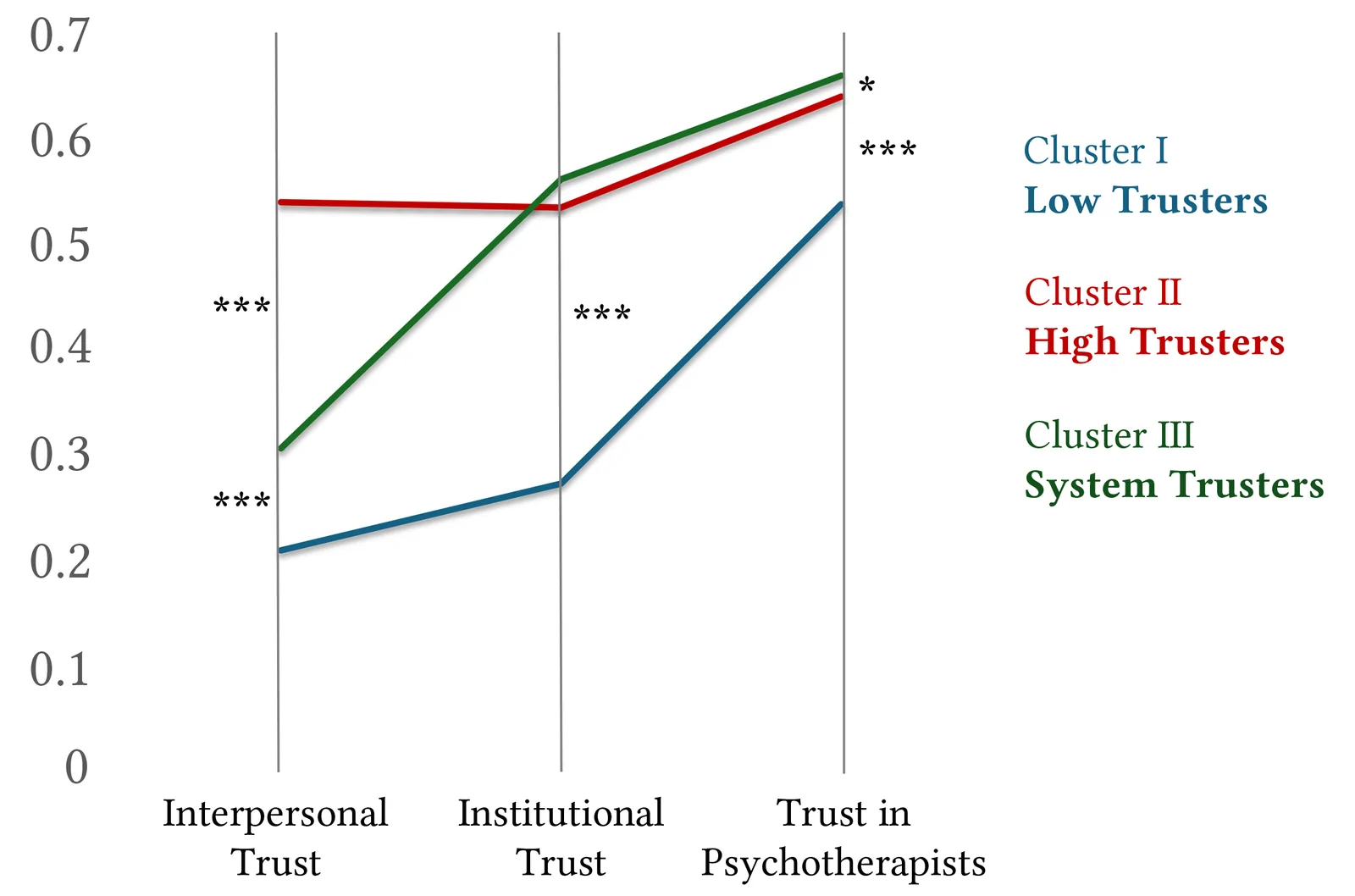
Trust Profiles per Cluster for Psychotherapy Patients *Note*. The mean scores per cluster were standardized: $\left(m_{Cluster}-Min\right)/\left(Max-Min\right)$. *p < .05. **p < .01. ***p < .001.

The hierarchical clustering resulted in a 3-cluster solution for those participants that have gone through psychotherapy. The trust-profiles for each cluster can be seen in Figure 1. Each cluster of the exploratory study design showed its own trust-profile. Based on the mean values of the three trust categories (trust in psychotherapists, institutional trust, interpersonal trust) the clusters 1-3 can be described the following way: *low trusters* (Cluster I; *n* = 391, 30%) with low scores for all trust measures, *high trusters* (*n* = 642, 49%) with highest interpersonal trust values and high trust values in psychotherapists and institutions and *system trusters* (*n* = 267, 21%) with highest trust scores in psychotherapists and high levels of institutional trust, but lower interpersonal trust values.

### Validation of Trust-Profiles

In order to assess the separation between the clusters, a discriminative analysis was examined and confirmed the three clusters. A total of two discriminant functions was obtained for the three cluster categories. Their Wilk’s λ values were 0.229 and 0.821, all reaching significant levels with *p* < .001. The eigenvalue of the first discriminant function was 2.582, which could explain 92.2% of the variance. The eigenvalue of the second discriminant function was 0.219 and explained 7.8% of the variance (Table 3). On the two discriminant functions, the structural load of institutional trust and interpersonal trust were highest (Table 4). The centroid of each cluster in the discriminant function is shown in Table 5. The reclassification results reached an accuracy rate of 87.2% (Table 6). In sum, the discriminant analysis indicated a clear separation between the three clusters but should be interpreted as a descriptive assessment rather than an independent validation of the cluster solution.

**Table 3:** Discriminant Function Significance Test for the Three Trust Clusters of the Hierarchical Cluster Analysis (HCA)

Function	Eigenvalues	Explained Variance	Cumulative Variation	$\rho^{2}$	Wilk’s λ
1	2.582	92.2%	92.2%	0.849	0.229***
2	0.219	7.8%	100%	0.424	0.821***

*Note.* Function 1 represents the first discriminant function, function 2 represents the second discriminant function.

***p < .001.

**Table 4:** Structural Loadings in Trust Dimensions for the Two Cluster Discriminant Functions

Variable	Cluster Discriminant Function	
	1	2
Interpersonal trust	0.933	-0.389
Trust in psychotherapists	-0.024	0.389
Trust in institutions	0.443	0.788

*Note.* Function 1 represents the first discriminant function, function 2 represents the second discriminant function.

**Table 5:** Functions at Group Centroids

Cluster	Functions at Group Centroids	
	1	2
Cluster I: Low Trusters	-2.134	-0.349
Cluster II: High Trusters	1.524	-0.164
Cluster III: System Trusters	-0.539	0.905

*Note.* Function 1 represents the first discriminant function, function 2 represents the second discriminant function.

**Table 6:** Classification Accuracy of the Three Cluster Solution

Cluster Category	Forecast Cluster			
	1	2	3	Total
1. Low Trusters	347 (89%)	0 (0%)	44 (11%)	391
2. High Trusters	8 (1%)	610 (95%)	24 (4%)	642
3. System Trusters	36 (14%)	55 (21%)	176 (66%)	267
Classification accuracy	87.2%			

*Note.* Function 1 represents the first discriminant function, Function 2 represents the second discriminant function, Function 3 represents the third discriminant function. Rows represent the original cluster assignments derived from the cluster analysis, whereas columns represent the cluster membership predicted by the discriminant analysis. Cell entries show the number of participants and the corresponding row percentages. Values on the diagonal indicate correctly classified participants; off-diagonal values indicate misclassifications. Overall, 87.2% of participants were correctly classified.

### Classification of Trust-Profiles

**Figure 2:**
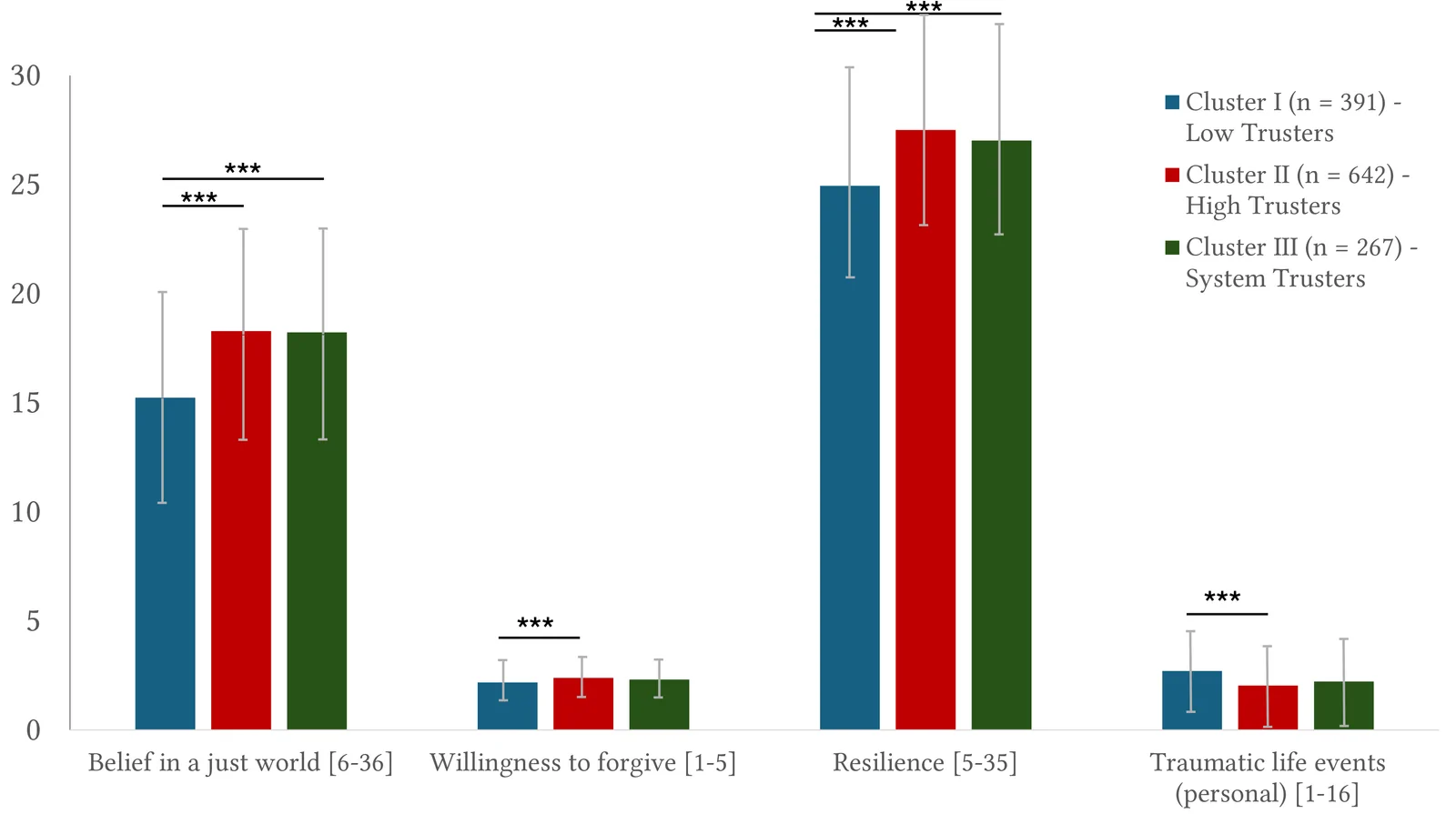
Belief in a Just World, Willingness to Forgive, Resilience and Trauma in the Three Trust Groups *Note.* Pairwise comparisons. *p < .05. **p < .01. ***p < .001.

**Figure 3:**
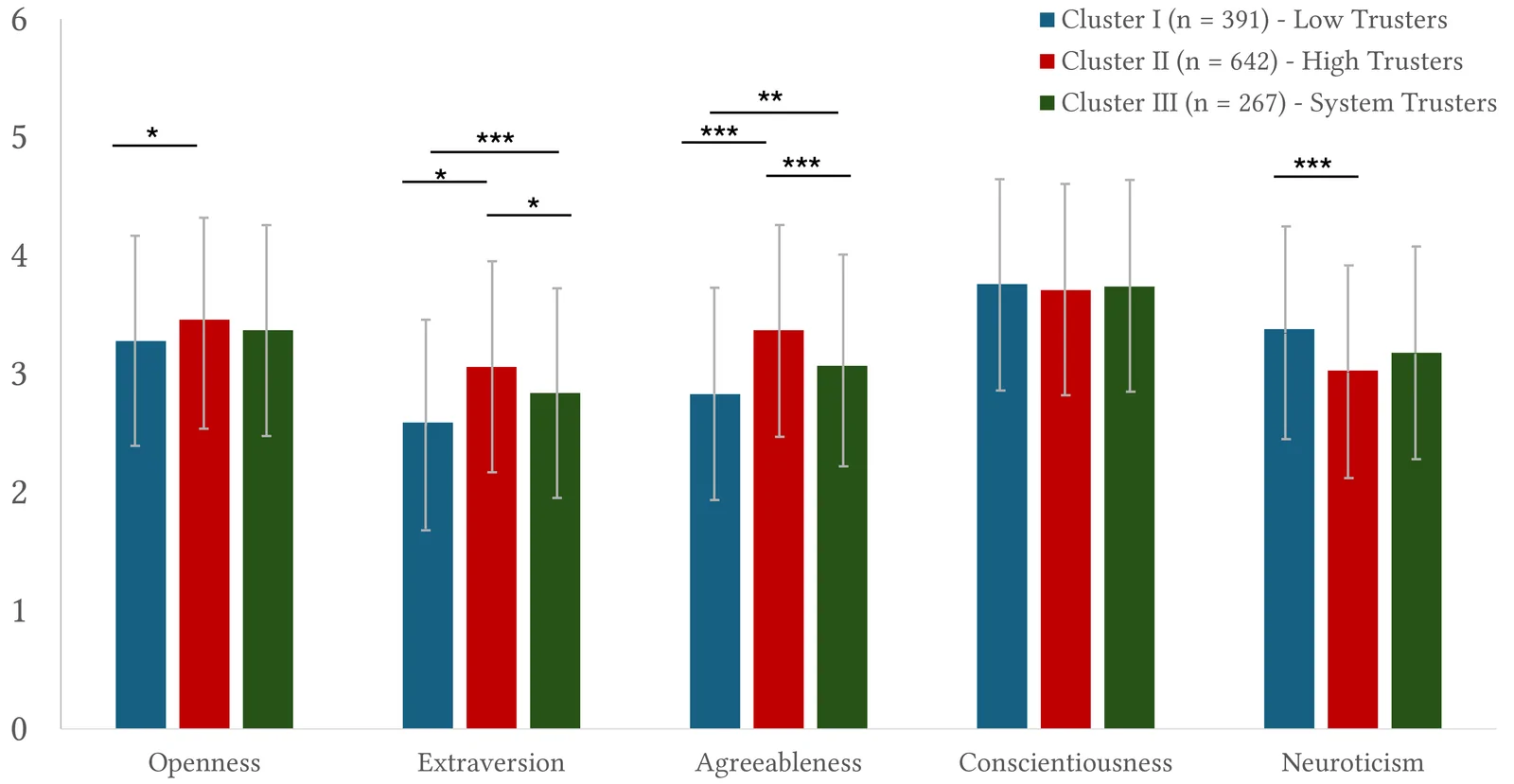
Personality Traits in the Three Trust Groups *Note.* Pairwise comparisons of the Big Five Personality Traits. *p < .05. **p < .01. ***p < .001.

The three trust groups differed according to all of the classification variables – expect conscientiousness (Table S3 Appendix): System trusters were less willing to forgive others, were less agreeable and extroverted, than the high trusters, who reached highest scores in these categories, but showed higher values than the low trusters. Both system trusters and high trusters more often believed in a just world. High trusters showed higher resilience scores and experienced less critical and potentially traumatic life events in comparison with the low trusters. The high trusters reached the highest score for openness and low trusters showed higher values of neuroticism (Figure 2 & Figure 3).

## Discussion

In this study, we investigated whether trust in psychotherapists is more closely linked to interpersonal or institutional trust. Our findings build on previous research, which predominantly considers trust in psychotherapists as an interpersonal construct (e.g., Ozawa & Sripad, 2013; Kassebaum, 2004). Our findings suggest that trust in psychotherapists is not solely associated with interpersonal factors but is also modestly connected with institutional trust, indicating that broader systemic attitudes may play a role in shaping patients' trust in psychotherapy. Based on HCA, we could identify three groups depending on the levels of institutional trust, trust in psychotherapists, and interpersonal trust: there are patients who show high levels in all three types of trust (high trusters), persons with low trust values in general (low trusters), and patients who trust the system and psychotherapists, but not others (system trusters).

While the clusters were derived exclusively from trust variables, subsequent analyses examining external variables revealed differences between clusters in terms of personality characteristics and life experiences. These findings provide preliminary support for the broader psychological relevance of the identified trust profiles. The three clusters of trust patterns exhibited partly different character traits and life experiences associated with psychotherapy usage and institutional trust. However, these findings should be interpreted with caution, as the cluster solution itself is based solely on trust variables. There may be several key starting points for psychotherapists in this context. The following considerations regarding therapeutic implications are speculative and should be interpreted with caution, as they are not directly tested in the present study. And it is important to distinguish between general trust in psychotherapists and trust in one’s own psychotherapist. The present study focuses on general trust, which reflects attitudes toward the profession rather than the quality of a specific therapeutic relationship. Firstly, it is important to recognize that, from the patient’s perspective, psychotherapists also represent institutions within which they work. Consequently, a trusting patient may also trust the system as a whole. For system trusters who trust psychotherapists but not others, additional support might be relevant to help them build new trusting relationships in private contexts, as observed in cases of psychosis patients (Ridenour et al., 2024) or young patients with suicide ideation (Hill et al., 2019). Interventions such as social skills training within the therapeutic context could be relevant for this group. In addition, there are patients who not only trust psychotherapists but also others and the political system – either as a result of psychotherapy or as a preexisting trait. For this group of high-trusters, potential risk factors for the emergence of psychiatric symptoms in the future may include impending critical life events or challenging circumstances, but at first, it is necessary to examine potential risk factors in longitudinal research. At the same time, practitioners may encounter patients who exhibit low trust overall (low-trusters). Low-trusters seem to be an especially vulnerable group: They experienced more traumatic life events, showed less resilience, were less open, extroverted, agreeable and reached higher levels of neuroticism. They were less willing to forgive others and believed less in a just world. For these patients, it could be relevant to foster trust within the therapeutic relationship. Additionally, the development of resilience factors, such as social participation, establishing effective coping strategies, and cultivating personal interests, may prove beneficial, too. These potential therapeutic implications should be interpreted with caution, as they are not directly tested in the present study and remain speculative.

Further investigations on psychotherapeutic relationships are warranted to prove distinct connections. Future research could examine whether cluster affiliation is associated with specific psychiatric outcomes and should take into account that trust in psychotherapists is associated with institutional trust. This is an important aspect regarding the conceptualization of trust in psychotherapists.

These findings align with the recommendations of Gaebel and colleagues (2014) to preserve trust in the healthcare system and psychiatrists. Our findings broaden the range of strategies for enhancing trust in psychotherapists and, in turn, may also improve the effectiveness of psychotherapy. We recommend the regular evaluation of trust in psychotherapy.

### Limitations

The representativeness of this study is limited due to the non-random sampling and the inherent characteristics of panel surveys. The results of these correlational analyses suggest the potential for the formulation of causal hypotheses regarding trust in psychotherapists, which should be investigated further. The analysis can only be described as exploratory. Given the diverse definitions of trust in psychotherapists, it cannot be ruled out that different questionnaires might yield varying cluster solutions. A combined analysis incorporating multiple trust questionnaires could provide further information and strengthen our findings. Furthermore, a limitation of this study is that it assesses general trust in psychotherapists rather than trust in a specific therapist, which may differ in important ways. Also, the concept of epistemic trust – the capacity to understand others’ and one’s personal behavior according to mental states, which is relevant in the psychotherapeutic relationship – was not measured (Fonagy & Allison, 2014). In addition, the identified clusters represent statistical groupings based on the included trust variables and should not be interpreted as clearly distinct psychological types. Besides, the data used is only based on self-reports. Furthermore, the experience of psychotherapy was quite broad: It included participants who are currently in therapy, have completed therapy, or have discontinued therapy. These experiences can vary and may be related to trust in psychotherapists. Finally, the results refer to the German healthcare system, which is characterized by relatively high institutional regulation and broad access to psychotherapeutic services. Given cultural differences, no generalizations can be made. These structural features may contribute to comparatively higher levels of institutional trust, which in turn could influence trust in psychotherapists. Research suggests that the relative importance of interpersonal versus institutional trust may vary across cultural contexts (e.g., Sikorski & Albrecht, 2025). Therefore, the generalizability of the present results to other countries and healthcare systems may be limited and should be examined in future cross-cultural studies.

### Conclusion

Trusting the psychotherapist may imply trusting the system: This perspective emphasizes the need for an adjustment of the conceptualization of psychotherapy as a relationship embedded within an institutional framework. In the therapeutic relationship, a third entity – the institution or system – plays a role alongside the patient and the psychotherapist. This underscores the importance of evaluating not only the patients’ trust in the psychotherapists but also their perception of the (healthcare) system as perceived by both the psychotherapist and the patient. This approach may help optimize the efficacy of psychotherapeutic treatments.

## Supplementary Materials

The Supplementary Materials contain the following items (for access, see Kaufmann et al., 2026S):

- Table S1. Sample description of the cross-sectional study (online)
- Table S2. Profiles of the three clusters of the hierarchical cluster analysis (HCA)

KaufmannM.
KasingerC.
HellerA.
BrählerE.
StraußB.
 (2026S). Supplementary materials to "Trusting the psychotherapist, trusting the governmental system and perhaps trusting the neighbor next door: Exploring the institutional nature of trust in psychotherapists and examining different groups of trusters in a large German patient sample"
[Appendix]. PsychOpen. 10.23668/psycharchives.22342


## Data Availability

The data that support the findings of this study are available from the corresponding author upon reasonable request.

## Preregistration

The study was not preregistered.

## Reporting Guidelines

This study was conducted in accordance with the JARS–Quant Guidelines.
